# Oxygen reductase origin followed the great oxidation event and terminated the Lomagundi excursion

**DOI:** 10.1016/j.bbabio.2025.149575

**Published:** 2025-12-01

**Authors:** Katharina Trost, Robert B. Gennis, John F. Allen, Daniel B. Mills, William F. Martin

**Affiliations:** a Department of Biology, Institute for Molecular Evolution, Heinrich Heine University of Duesseldorf, 40225, Duesseldorf, Germany; b Department of Chemistry, University of Illinois Urbana-Champaign, USA; c Research Department of Genetics, Evolution and Environment, University College London, UK; d Department of Earth and Environmental Sciences, Paleontology & Geobiology, Ludwig-Maximilians-Universität München, 80333, Munich, Germany

## Abstract

The history of Earth’s atmospheric oxygen is a cornerstone of evolutionary biology. While unequivocal evidence for an increase in atmospheric O_2_ marks the Great Oxidation Event (GOE) roughly 2.4 billion years ago, evidence underlying proposals for pre-GOE O_2_ accumulation is debated. Here we have investigated the distribution of genes for oxygen reductases, the enzymes that consume O_2_ in respiratory chains, across independently generated molecular timescales of prokaryotic evolution. The data indicate that cytochrome *bd*-oxidases, heme-copper oxidases and alternative oxidases arose in the wake of the GOE ca. 2.4 billion years ago, after which the genes were subjected to abundant lateral gene transfer, a reflection of their utility in redox balance and membrane bioenergetics. The data lead us to propose a straightforward four-stage model for O_2_ accumulation surrounding the GOE: (i) Negligible O_2_ existed prior to the GOE. (ii) Cyanobacterial O_2_ production started at the GOE, yet was capped at 2 % [*v*/v] atmospheric O_2_, the threshold at which cyanobacterial nitrogenase is inhibited by O_2_. (iii) Production of 0.02 atm of O_2_ (2 % [*v*/v]) at the GOE buried roughly the entire atmospheric CO_2_ inventory, causing sudden enrichment of ^13^C in dissolved inorganic carbon (the Lomagundi ^13^C anomaly), through RuBisCO isotope discrimination, without atmospheric O_2_ exceeding 2 % [*v*/v]. (iv) High atmospheric ^12^C at the end of the Lomagundi excursion marks the origin of oxygen reductases, their rapid spread via function in respiratory CO_2_ liberation, and the onset of equilibrium between photosynthetic O_2_ production and respiratory O_2_ consumption at 2 % atmospheric O_2_.

## 1 Introduction

Molecular oxygen, O_2_, accumulated in the Earth’s atmosphere starting ~2.4 billion years ago (Ga) during the Great Oxidation Event or GOE, as documented by several lines of evidence [1–3]. Among them, heavy stable carbon isotope ratios, δ^13^C (δ^13^C = [(^13^C/^12^C)_sample_/(^13^C/^12^C)_standard_] - 1), in sedimentary rocks serves as a proxy for increased organic carbon burial, which enable the persistence of photosynthetically derived O_2_ in Earth’s atmosphere [4,5]. Another important indicator of Earth’s atmospheric oxygenation are measurements of mass-independent sulfur fractionation, or MIFs, which put a strict upper limit of 10^−6^ present atmospheric level (PAL), or 10^−7^ atm, prior to the GOE [6]. There are, however, reports that traces of atmospheric O_2_ accumulation, called “whiffs,” commenced slightly earlier than the GOE [7,8]. Those reports have been challenged, however, as newer findings indicate that the whiffs are caused by later oxidation of 2.45 Ga sediment samples that were deposited in the absence of O_2_ [9]. Anbar et al. [10] responded to that report and [11] responded in return. There are also reports that synthesis of O_2_ from sand could have generated O_2_ pre-GOE [12–14], but the proposed mechanism involves the synthesis of H_2_O_2_, which is too reactive to have contributed to O_2_ accumulation on an atmospheric scale [15,16]. The half-life of H_2_O_2_ is only 0.7 s in the presence of Fe^2+^ [15], which would preclude its role as a source of environmental O_2_ or as a possible precursor to H_2_O in the evolution of the oxygen evolving complex (OEC) of photosystem II [17]. There are also reports that ocean floor manganese nodules can synthesize O_2_ [18], but the nodules in question are formed and deposited with the help of O_2_, rendering any such contribution to pre-GOE O_2_ production unlikely at best.

Several molecular phylogenetic studies of oxygen-utilizing enzymes [19–23] or enzymes related to oxygen-utilizing pathways [24,25] infer an origin of oxygenic photosynthesis prior to the GOE on the basis of molecular clocks. But such studies entail the assumption of strict vertical inheritance for prokaryotic genes, that is, no lateral gene transfer (LGT) or at most one LGT from an unknown extinct donor [24], whereby it is known that all prokaryotic genes studied to date have been subjected to multiple LGTs during evolution [26], including—and in particular—O_2_-dependent enzymes, which are among the most frequently transferred genes in prokaryotes [16]. Furthermore, molecular clock studies require the use of geochemical and paleontological calibration points, whereby there is no agreement as to what constitutes reliable evidence for pre-GOE O_2_. For example, Davin et al. [22] calibrated their trees assuming that the Fe and U-Th-Pb isotope signatures reported by Satkoski et al. [27,28] represent a hard minimum age for photosynthetic O_2_ production by 3.2 Ga, 800 MY before the GOE, whereby reports using chromium isotopes to infer pre-GOE O_2_ at 3.0 Ga [29] were challenged based on evidence for later oxidative weathering [30]. Isotope-independent biomarker data supporting the existence of cyanobacteria at 2.7 Ga [31] turned out to be contamination from younger rocks [32]. Using post-GOE prokaryotic fossils as calibration points [33] dated the origin of cyanobacteria to roughly 3 Ga, but no fossil cyanobacteria of that age are known, and fossils once thought to be 3.5 Ga cyanobacteria [34] turned out to be abiotic structures of hydrothermal vents [35]. Finally, the molecular clocks of Jablońska & Tawfik [23] inferred evidence for O_2_ before the GOE were not calibrated on geochemical data but using published molecular clocks. If we recall that MIFs put a strict upper limit for O_2_ of 10^−7^ atm prior to the GOE [6,36], all reports of pre-GOE O_2_ carry the caveat that pre-GOE O_2_ production was restricted to a particular local environment, and never accumulated in the atmosphere.

It is possible that, prior to the GOE, soluble Mn served as an evolutionary precursor substrate for the primordial oxygen evolving complex prior to the use of water as electron donor, but in a process that does not produce O_2_ [37,38]. There is no question that O_2_ became environmentally and physiologically relevant at the GOE [6]. What if there was no rudimentary or locally restricted O_2_ production before the GOE, which is possible [39]? What if the GOE is telling it like it was? In a straight-forward read of the geochemical record, the appearance of biologically relevant amounts of O_2_ on Earth corresponds 1:1 with the GOE. In that case, the GOE marks the maximum age of O_2_ respiration by prokaryotes because without the substrate (O_2_), the O_2_-reducing enzymes of respiratory chains [40,41], and other O_2_ dependent enzymes [16] could have no selectable O_2_-dependent function. This line of reasoning—that the GOE is the calibration point for the origin of O_2_-dependent enzymes—is almost entirely absent in the molecular-based literature on O_2_ history, and no molecular dating studies, except of Soo et al. [42], to our knowledge have suggested an origin of O_2_ dependent enzymes subsequent to the GOE, that is, molecular dating studies consistently date the origin of O_2_ pre-GOE.

The GOE is not, however, a simple event. The end of the GOE is accompanied by the Lomagundi-Jatuli Excursion (LJE, also called the Lomagundi excursion), the largest event of elevated, seawater-derived δ^13^C values over the last 3.5 billion years [43,44]. During the LJE, δ^13^C values increased to roughly +5 to +10 ‰, indicating, at face value, massive primary production and carbon burial, which under standard geochemical models [3,4,45] corresponds to massive O_2_ production (between 12 and 22 times the present atmospheric reservoir; [4]). There is no consensus about the interpretation of the LJE. It could indicate a global event or a series of coastal, shallow water events [45–48] that lasted approximately 100 to 250 Ma, from 2.3 to 2.0 billion years ago [46]. Using standard atmospheric models [3,4,45], the magnitude of δ^13^C enrichment at the LJE would imply that O_2_ rose from zero pre-GOE to levels greatly exceeding the value of 21 % (*v*/v) in today’s atmosphere. There are, however, reasons to doubt that standard atmospheric models apply to the LJE, leaving the cause and impact of the δ^13^C anomaly during the LJE, in terms of O_2_ levels, an open question [48].

Following the LJE, δ^13^C values fall to levels indicating roughly 1–10% of present atmospheric O_2_ levels (PAL) for almost 2 billion years until the appearance of land plants [49–51]. Geochemists debate the reasons for that continued phase of low oxygen [52–57], but the simplest explanation is biological, and enzymatic, in that nitrogenase is inhibited by O_2_, and that inhibition limits cyanobacterial growth and O_2_ production, on a global scale, until O_2_ production by land plants set in ~500 MY ago [16,58–61]. During that time, oxygen reductases arose and spread, also into the eukaryotic lineage via the origin of mitochondria [60,62,63].

**Fig. 1:**
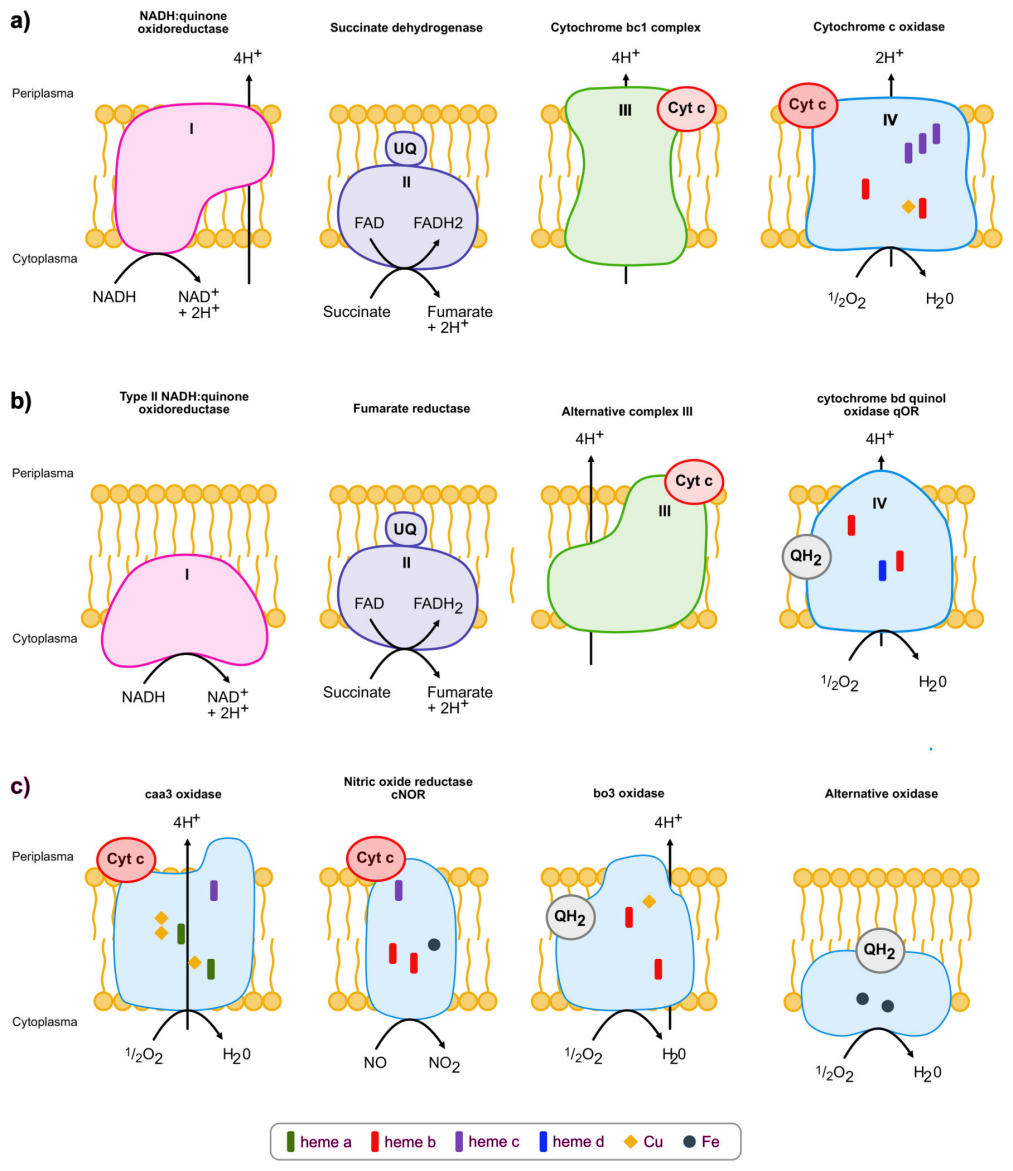
Components of the respiratory chain and different types of oxygen reductases. A) components of the classical respiratory chain and b) alternative complexes of the respiratory chain. In c) different types of oxygen reductases are shown including the caa3 oxidase (HCO), the nitric oxide reductase cNOR (HCO), the bo3 oxidase (HCO) and the alternative oxidase (AOX).

On the modern Earth, O_2_ consumption by oxygen reductases roughly equals O_2_ production [64,65]. Without biological O_2_ consumption through respiratory terminal oxidases, O_2_ would rise to levels that promote spontaneous combustion in forests. There are four basic types of oxygen reductases that maintain O_2_ at 21 % v/v including the cytochrome *bd*-type oxygen reductases (bd), the heme-copper oxygen reductases (HCO), the alternative oxygen reductases (AOX) and the plastoquinol terminal oxidase (PTOX) (Fig. 1c) [40–42,66–70]. The bd-, HCO-types of reductases are known to be highly affected by LGT even between domains (Bacteria and Archaea) and thus are distributed over a wide range of prokaryotes [16,40,41,66,68–71]. The alternative oxygen reductases (AOX) are present in eukaryotes and in marine bacteria [68,72] while PTOX can only be found in photosynthetic organisms including higher plants, alga, diatoms and Cyanobacteria [72–74]. AOX and PTOX are membrane bound quinol reductases but have no role in energy conservation, solely serving the function of maintaining redox balance and avoidance of over reduced quinol pool in the bioenergetic membrane instead [75–77]. The *bd*-type and HCO oxygen reductases conserve energy in the form of proton gradients [40,41] and are likely no older than the GOE [42], having arisen in oxic environments [16]. The HCO family includes the nitric oxide (NO) reductases, which are evolutionarily derived from O_2_ oxidase ancestors [40,41,66,68,69,78].

The timing of oxygen reductase origin is an unresolved issue, though the oxygen affinity of *bd*-type, HCO and AOX reductases suggest a sequence of order in their evolution: While *bd*-type oxidases have high oxygen affinity, typically occurring in environments with low O_2_-levels, the affinity of HCO and AOX and PTOX oxygen is low, requiring O_2_-rich environments for activity [79,80]. Here we investigate the timing of oxygen reductase origin and their spread across prokaryotic lineages by mapping their distributions across time-calibrated phylogenetic trees [81]. Our approach presents a radical departure from previous studies in that (i) we accept the date of the GOE as the earliest possible time of oxygen reductase origin and function, (ii) we accept the existence of LGT in oxygen reductase evolution, and (iii) we use a non-controversial molecular dating scheme for prokaryotic evolution that was generated by third parties and not for the purpose of dating oxygen reductase evolution. The findings highlight physiology surrounding the GOE and uncover a biological model that can account in a surprisingly direct manner for the δ^13^C isotope anomaly at Lomagundi-Jatuli excursion as the product of a single cyanobacterial enzyme.

## 2 Methods

### 2.1 Prokaryotic time tree

The prokaryotic dated tree of life was obtained from Mahendrarajah et al. [81]. It comprises 863 strains including 350 bacterial, 350 archaeal and 163 eukaryotic genomes.

### 2.2 Balanced prokaryotic RefSeq dataset

The prokaryotic sequences were downloaded from the Reference Sequence Database (RefSeq) release 223 in May 2024 from the National Center for Biotechnology Information (NCBI; [82]) including 41,210 prokaryotic genomes. To avoid any phylogenetic bias, a balanced sample was generated using the biggest archaeal genome per species and the biggest bacterial genome per family. Additionally, 11 genomes with less than 1000 proteins were filtered out and 9 genomes from organisms that have no cytochromes and which were found by Rosenbaum and Müller [83] were added. In total, the balanced dataset comprises 953 genomes including 552 bacterial and 401 archaeal genomes.

### 2.3 Oxygen reductases proteins

The set of 265 *bd*-type oxygen reductase sequences were obtained from Murali et al. [40]. From Murali et al. [41] 35,352 heme-copper oxygen reductase proteins were downloaded. A set of group-specific consensus sequences for alternative oxygen reductase proteins were downloaded from Weaver & McDonald [84] including 21 sequences of eukaryotic and prokaryotic groups. The plastoquinol terminal oxidase was taken form species *Anabaena cylindrica* with the accession number AFZ5900.1, downloaded from NCBI in December 2024.

### 2.4 Heme biosynthesis and cytochrome b protein sequences

The heme biosynthesis proteins for the protoporphyrin pathway were downloaded from RefSeq Release 227 (NCBI, [82]). The proto-porphyrinogen oxidase (PgoX) was obtained from the species *Staphylococcus aureus* and all other protoporphyrin pathway proteins were obtained from species *Klebsiella Pneumniae*. The coproporphyrin pathway proteins were all from *Staphylococcus aureus* and the proteins from the siroheme pathway proteins were from *Methanosarcina barkeri*.

The cytochrome *b* proteins corresponding to the HdrDE complex from *Methanosarcina barkeri* were downloaded from RefSeq Release 227 (NCBI, [82]). As no complete sequences for the proteins of the VhtACG complex could be downloaded from RefSeq, we used hmmer profiles from InterPro [85].

### 2.5 Presence and absence of oxygen reductase proteins within a dated tree of life

The 265 proteins from *bd*-type oxygen reductase, the 35,352 heme-copper oxygen reductase proteins, the 21 alternative oxygen reductase proteins and the plastoquinol terminal oxidase sequence [40,41,84] were blasted against the balanced prokaryotic RefSeq dataset using Diamond version 2.1.8 [86]. Hits with an e-value ≤10E^-10^ and local identity ≥25 % were retained and cross-checked by protein annotation. Taxa corresponding to strains present in the remaining hits were colored in the dated tree of life using Interactive Tree of Life (iTOL v6, [87]) and the most ancient possible gene origins were calculated based on the sum of branch length of the deepest colored nodes in the dated tree of life. For phylogenetic tree analysis python ETE3 [88] was used.

### 2.6 Presence and absence of heme biosynthesis and cytochrome b proteins in Methanogens and Halophiles

All heme biosynthesis proteins and proteins of the HdrDE complex including cytochrome *b* were blasted against the genomes of Methanobacteria, Methanococci, Methanopyri, Methanomicrobia, Methanoliparia, Methanonatronarchaeia, Archaeoglobi, Thermoproteota and Halobacteria using Diamond version 2.18 [86]. Hits with an e-value ≤10E^-10^ and local identity ≥25 % were retained and cross-checked by protein annotation. The resulting best hits per protein were used as a proxy for presence or absence within the genome.

HMMER profiles of the VhtACG complex were searched against the genomes of Methanobacteria, Methanococci, Methanopyri, Methanomicrobia, Methanoliparia, Methanonatronarchaeia, Archaeoglobi, Thermoproteota and Halobacteria using HMMER version 3.3.2 (hmmer.org). Only hits with an e-value ≤10E^-10^ were retained and cross-checked by protein annotation. The best scoring hit per genome was used to infer presence or absence within the genome.

### 2.7 Monophyly of possible origin groups within oxygen reductase protein trees

Best blast hits per RefSeq genome were defined from the hits generated by the Diamond *blastp* search between reductase proteins and balanced RefSeq dataset for each oxygen reductase (see Taxonomic annotation of oxygen reductase proteins). From these, multiple alignments were made using MAFFT linsi v7.505 [89] and phylogenetic trees were generated using RAxML version 8.2.12 [90] under the PROT-CATWAG model. Groups of taxa corresponding to the most ancient possible gene origins were colored within the protein trees and monophyly of these groups were checked using python ETE3 and iTOL v6 [87,88]. Lateral gene transfer events per group and oxygen terminal oxidase were calculated by subtracting one from the number of clades present in the protein tree since one clade has to be the origin and all others are LGTs. To obtain a number of LGT events per terminal oxygenase the values for every group were summed up.

### 2.8 Statistical tests

Kernel density estimations were made for the distributions of origins of *bd*-type, HCO and AOX reductases. All statistical tests were performed using python. Kolmogorov-Smirnov test was used to compare the distribution of origin ages.

## 3 Results

### 3.1 Occurrence of oxygen reductases across prokaryotes

**Fig. 2:**
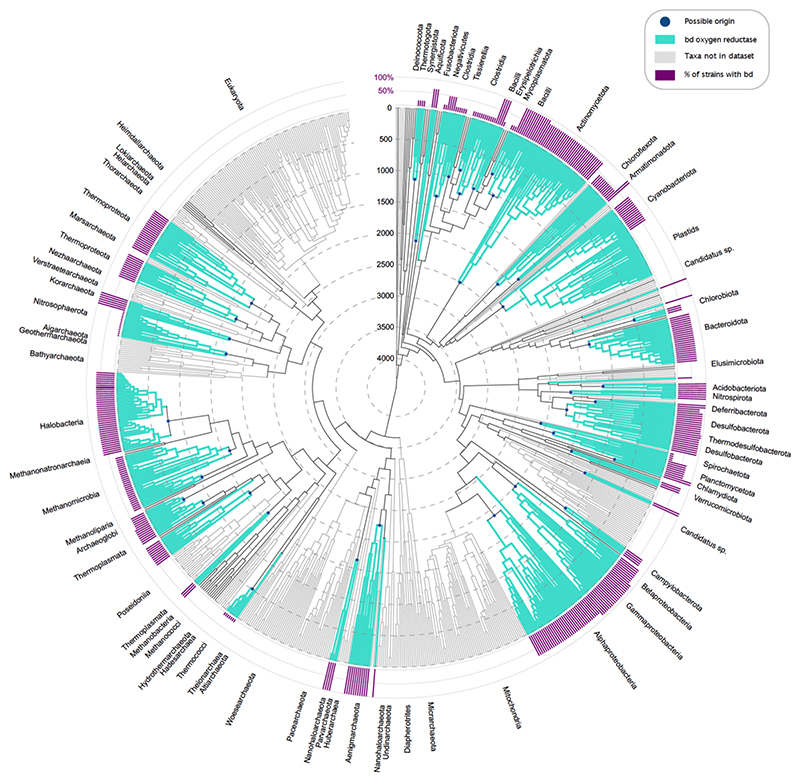
Occurrence of *bd*-type oxygen reductase in a dated tree of life. Branches in the dated tree of life obtained from Mahendrarajah et al. [81] are colored according to the presence (turquoise) or absence (gray) of *bd*-type oxygen reductase. Eukaryotes were not included in the analysis and are therefore colored in higher gray tones, as are taxa that were not present in the comparative dataset. Dark blue dots at nodes represent possible origins of *bd*-type oxygen reductase. Purple bars represent the percentage of strains within the taxa that have reductases. (For interpretation of the references to colour in this figure legend, the reader is referred to the web version of this article.)

**Fig. 3:**
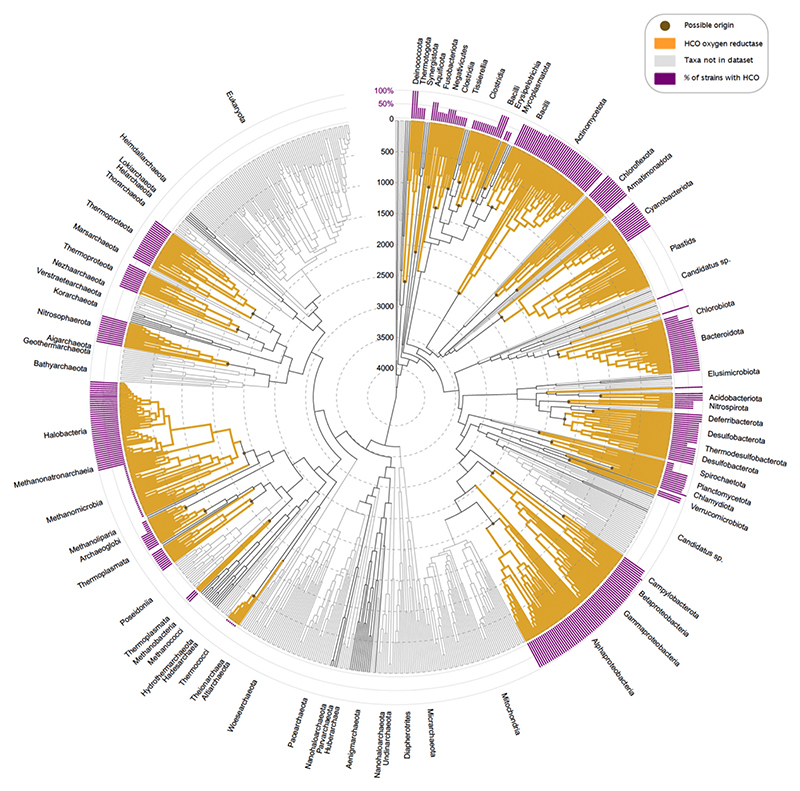
Occurrence of HCO oxygen reductase in a dated tree of life. Branches in the dated tree of life obtained from Mahendrarajah et al. [81] are colored according to the presence (yellow) or absence (gray) of HCO oxygen reductase. Eukaryotes were not included in the analysis and are therefore colored in higher gray tones, as are taxa that were not present in the comparative dataset. Brown dots at nodes represent possible origins of HCO oxygen reductase. Purple bars represent the percentage of strains within the taxa that have reductases. (For interpretation of the references to colour in this figure legend, the reader is referred to the web version of this article.)

To date the four types of oxygen reductases we used the dated phylogenetic tree with geological time spans as branch lengths constructed by Mahendrarajah et al. [81]. Based on diamond *blastp* [86] searches between protein sequences of *bd* [40], HCO [41], AOX [84] and PTOX reductases and a balanced prokaryotic genome dataset, we colored leaves and corresponding clades of taxa with *bd*, HCO or AOX and PTOX reductases sequences in the phylogenetic time tree (Figs. 2–3, Supplemental Figure 3). Leaves and clades corresponding to eukaryotes are colored in light gray since they were not part of the analysis, as well as taxa that were not present in the balanced prokaryotic dataset and therefore cannot be hit by our blast, as these taxa mainly correspond to metagenomic assemblies (MAGs) that are not represented in our balanced prokaryotic dataset.

Cytochrome *bd* reductases are common in Actinomycetota, Bacilli, Pseudomonadota and Halobacteria and less abundant in Chlorobiota, Clostridia, Fusobacteriota, Spirochaetota Mycoplasmatota, Nitrososphaerota, Thermococci and Thermotogota (Fig. 2). This distribution is consistent with previous studies [40,66], with the exception of the occurrence of *bd* in Thermotogota, where it is however only present in one of the five possible strains (Supplemental Table 1).

HCO reductases are more common in the current data than *bd* oxidases or alternative oxidases (AOX and PTOX). They are distributed across almost all taxonomic groups except for smaller archaeal and bacterial groups including Heimdallarchaeota, Korarchaeota, Nanohaloarchaeota, Aenigmarchaeota, Mycoplasmatota and Synergistota (taxonomy of NCBI as of January 2023). Additionally, we found isolated cases of blast hits for HCO proteins in methanogens, yet only in four strains of Methanomicrobia and one of Methanonatronarchaeia (Fig. 3, Supplemental Table 1). Because (i) all HCOs contain heme and (ii) methanogens are not able to synthesize heme except of some species corresponding to Methanosarcinales, for example *Methanosarcina barkeri* [41,91], we performed Diamond *blastp* searches of heme biosynthesis proteins against methanogens and Halobacteria, to see whether the presence of HCO reductases in Methanomicrobia and Methanonatronarchaeia could be chance similarity or the result of an LGT that does not generate a functional protein (that is, a component of the accessory genome). Among methanogens, only strains of Methanosarcinales encoded a full heme biosynthesis pathway (Supplemental Fig. 1), 96 % of strains of Methanosarcinales in our dataset encoded the three key proteins for the alternative siroheme pathway (Supplemental Table 2). Additionally, we checked whether the sampled methanogens possess the VhtACG and HdrDE protein complexes, which are involved in energy conservation of species of Methanosarcinales and are known to contain cytochrome *b* [92,93]. The complete VhtACG and HdrDE protein complexes were only present in some strains of Methanosarcinales and Methanonatronarchaea (Supplemental Fig. 2). However, the VhtC protein, which includes cytochrome *b*, is also present in Halobacteria, Archaeoglobi, Thermoproteota and Methanocellales. The other cytochrome-containing protein HdrE was only detected in Methanosarcinales (all), one strain of Methanomicrobiales, and the lone Methanonatronarchaeal strain. Based on the absence of heme biosynthesis cytochrome *b* containing protein complexes VhtACG and HdrDE, the occurrence of a putative HCO in the Methanotrichales strain of Methanomicrobia is probably attributable to sequence similarity to other oxidases. Although all methanogens known are strict anaerobes, HCO reductases can in principle be present in the three remaining Methanosarcinales strains, though we found no reports of their possible expression or function. Outside the methanogens, HCO reductases are otherwise well known to be present throughout the tree of life, with involvement in both aerobic and anaerobic respiration [41,78,79,94].

Alternative oxidases including AOX, an additional terminal oxidase in mitochondrial electron transport, and PTOX, the plastoquinol terminal oxidase which is the relative enzyme of the photosynthetic electron transport chain [95] are less common in prokaryotes [68,74,84,96]. Consistent with previous analyses, we found AOX reductases only in Pseudomonadota, specifically Alpha-, Beta- and Gammaproteobacteria (Supplemental Fig. 3; [68,84,96]) and PTOX sequences in Cyanobacteriota (Supplemental Fig. 3; [74]). One AOX sequence was also found in Cyanobacterium *Picosynechococcus*, but as this is likely to reflect sequence similarity between AOX and PTOX [74], we excluded this genome for further analysis with AOX.

### 3.2 Timing the origins and spread of oxygen reductases

**Fig. 4:**
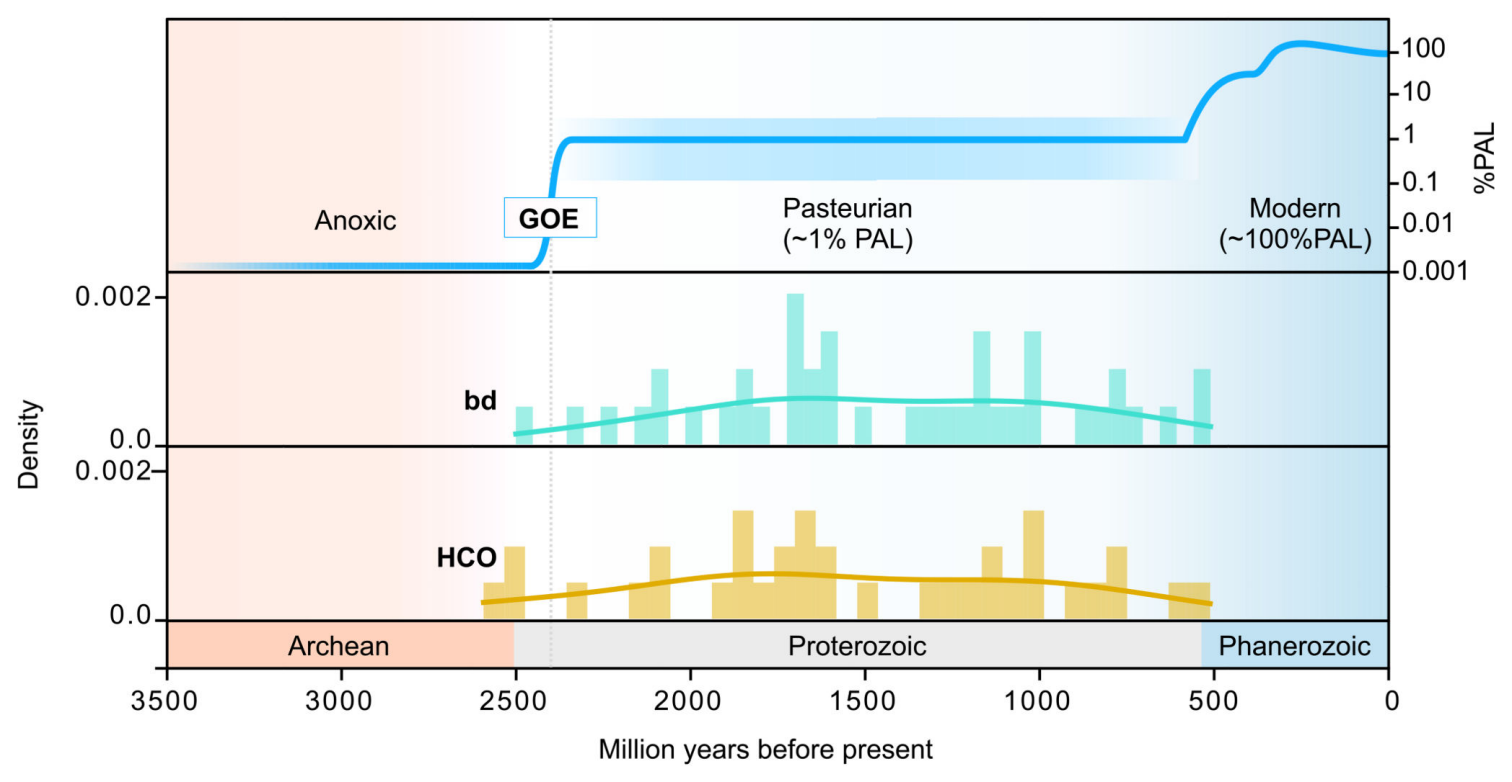
Distribution of ages per group on geological timescale. Distribution of ages per possible origin (group) within the dated tree of life for the bd-type (turquoise) and HCO (yellow) oxygen reductases. The age [Ma] per group is shown on the x-axis and the corresponding kernel density function (KDE) is placed over the corresponding distribution. The distributions of AOX and PTOX can be found in Supplementary Fig. 4. (For interpretation of the references to colour in this figure legend, the reader is referred to the web version of this article.)

To estimate the time of origin for each oxygen reductase, we used the deepest node for each colored clade and calculated the age of the possible origin by summing up the branch lengths. This conservatively delivers a maximum age for the respective reductases in each clade. For *bd* oxygen reductase we identified 41 possible origins (independent clades) and for HCO 33 possible origins. The AOX and PTOX reductases are the least frequently distributed across the prokaryotic time tree, reflecting only two possible origins for AOX and one origin for PTOX (Supplemental Table 1). The timing of the (earliest) origin of *bd* oxidases and members of the HCO family within a given prokaryotic clade can, with many caveats, be read directly off the timed tree generated by Mahendrarajah et al. [81]. We plotted the distribution of ages for each possible origin on a geological timespan (Fig. 4 and Supplemental Fig. 4). For each distribution except of PTOX (due to the sample size of one) we calculated a Kernel Density Estimation (KDE) to estimate the probability distribution of ages of origins over the entire time period.

What does the age of a *bd* clade or an HCO clade indicate? The *bd* oxidases are all related in sequence, structure and function, they descend from a single common ancestor. We observe, for example, 41 clades of prokaryotes that harbor *bd* oxidase genes. At the one extreme, these 41 clades could be the result of a single *bd* oxidase gene origin in the common ancestor of bacteria and archaea followed by differential loss. This kind of strictly vertical reasoning places all proteins present in some bacteria and some archaea in the last universal common ancestor LUCA. It would place the age of *bd* oxidases at roughly 4 billion years and entail their persistent presence, without oxygen, throughout diverse basal branches in the tree for at least 1.8 billion years, up until the GOE. This kind of “no LGT” scenario calls for geological sources of sustained O_2_ production prior to the GOE—controversial sources [12–14,18]—that are however not documented in the geological record, because the first uncontested appearance of biologically useful (respirable) amounts O_2_ on Earth *is* the GOE. A “no LGT” model also calls for explanation of why other studies find evidence for substantial amounts of LGT in the evolution of *bd* oxidases and all other prokaryotic genes [40,42,66,84,97].

The other extreme is that only *one* lineage among the 41 *bd* containing clades invented *bd* oxidases and that all other 40 clades are the result of subsequent lateral transfers from the original inventing clade or from secondary spread. That would entail a great deal of LGT in bd oxidase evolution, consistent with recent studies [16]. It would mean that the first origin of *bd* oxidases occurred roughly 2.5 billion years ago (the oldest *bd* origin in the tree, in Actinomycetota), and very close to the GOE (2.4 Ga), within the limits of accuracy on the Mahendrarajah et al. [81] tree. It would entail no requirements for the existence of respirable oxygen prior to the GOE. In fact, this extreme (one origin, 40 LGTs) fits the observations from gene evolution and a straight reading of the geochemical record well, with no need for corollaries.

The ages of the 41 *bd* origins are distributed between 2500 and 510 Ma ago with only one origin before the time of the GOE (Fig. 4, Supplemental Table 1). Since, for the purposes of this paper, we posit that there was no oxygen before the GOE [15,16], the possible origins before the GOE contributing to Actinomycetota (age origin Actinomycetota = 2501 Ma) is likely a result from LGT into the Actinomycetota lineage. All other possible origins are distributed at timespans after the GOE with Cyanobacteriota having the oldest origin (the age of Cyanobacteriota is 2325 Ma in the calibration of Mahendrarajah et al. [81]) with Archaeoglobi (623 Ma), Thermococci (512 Ma) and Chlorobiota (510 Ma) (see Supplemental Table 1 for a list). The KDE for *bd* shows a peak of origins around 1600–1700 Ma which indicates a large number of *bd* oxidase origins in different lineages (spread via LGT) during this time span (Fig. 4). In comparison, the average age of *bd* origins is 1430 Ma, slightly lower than the peak around 1600–1700 Ma (Supplemental Table 3). Due to LGT, many origins of smaller taxonomic groups could affect the average age of origins and thus easily distort it to a lower average age. Still, the peak at 1600–1700 Ma is within the range of average origin age ± one standard deviation (STD, Supplemental Table 3).

The distribution of ages of HCO reductase origins is similar to that of *bd*-type reductases (Kolmogorov-Smirnov Statistic = 0.111, *P* = 0.945). HCO origins are distributed between 512 and 2593 Ma with two possible origins before the GOE (Fig. 4, Supplemental Table 1). These two origins correspond to the taxa Deinococcota, Thermotogota (age = 2593 Ma) and Actinomycetota (age = 2501 Ma). After that, the next origin is located in Beta-, Gamma- and Zetaproteobacteria (age = 2477; Supplemental Table 1) which is consistent with a previous study, suggesting that HCO may originate in basal lineages of Pseudomonadota [98]. Taxa including late possible origins for HCO reductase are Chlamydiota, Archaeoglobi and Thermococci (age origin Chlamydiota = 790 Ma, age origin Archaeoglobi = 623 Ma, age origin Thermococci = 512 Ma; Supplemental Table 1). The KDE has a peak of origin frequency at 1700–1800 Ma, as for *bd*-type reductases, and a second peak around 1000 Ma (Fig. 4). The average age of all HCO origins is at 1523 Ma, again slightly lower as the peak within the KDE. Noticeable for both distributions and KDEs of *bd*-type and HCO reductases is that the origins only occur within the timespan of the Pasteurian billion (also called the boring billion [50,99,100], between 1800 and 800 Ma. Thus, the data indicate that oxygen reductases arose and were spread across prokaryotes (i) after the GOE and (ii) during the time period of low oxygen in Earth history (the Pasteurian billion). Similar results were found for AOX and PTOX (Supplemental Figs. 3–4, Supplemental Table 1).

### 3.3 Oxygen reductases are strongly affected by LGT

**Fig. 5:**
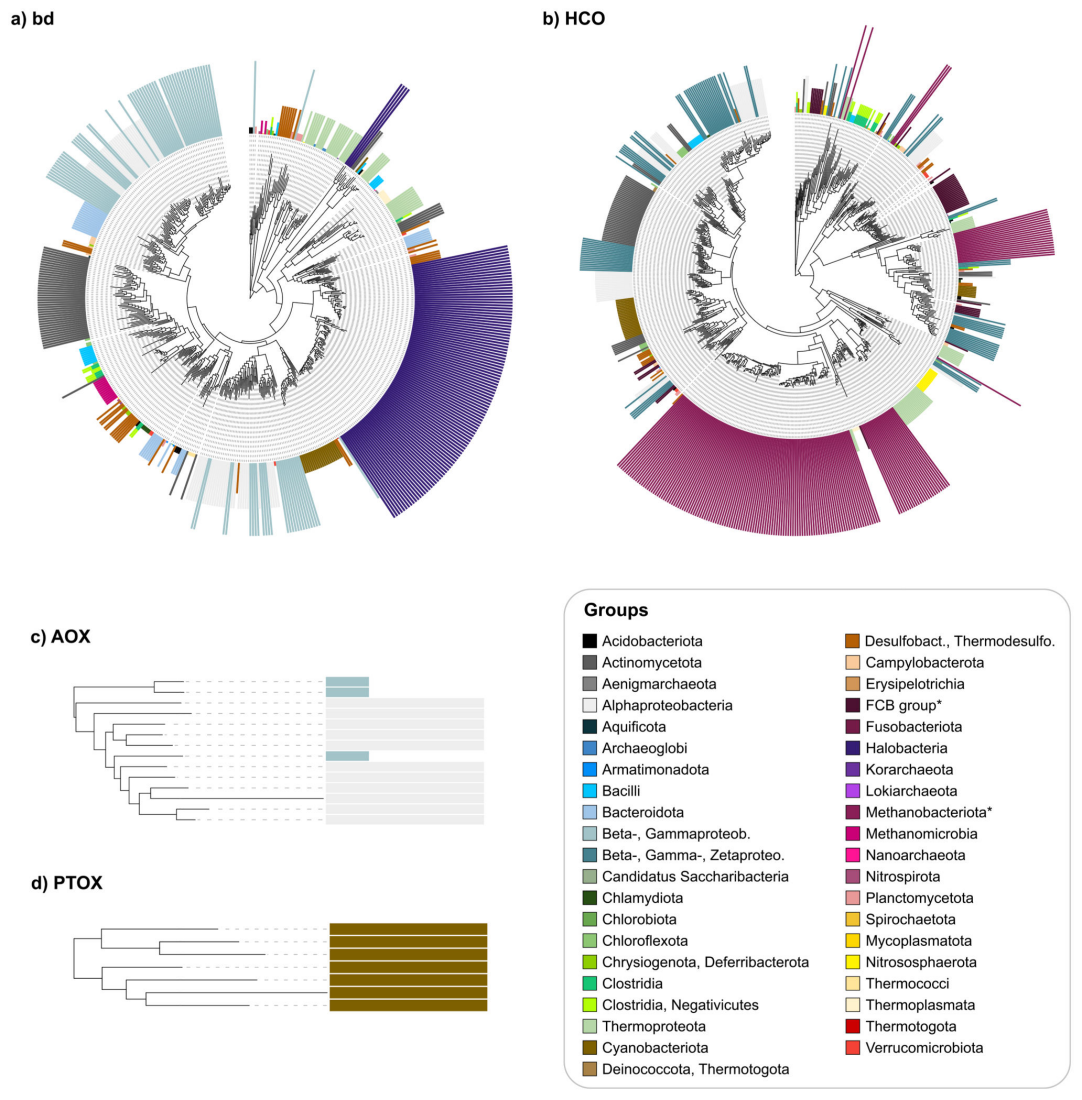
Size and included taxa of groups within the protein trees of bd-type, HCO, AOX and PTOX oxygen reductase. The leaves of the protein trees for bd-type (a), HCO (b), AOX (c) and PTOX (d) oxygen reductases are colored based on their affiliation to groups, found in the dated tree of life. The sizes of the colored strokes represent the number of strains present in the group. The corresponding taxa included in every group are shown in the right bottom box. *FCB group includes Bacteroidota, Balneolota, Chlorobiota, Rhodothermota and *Methanobacteriota includes Methanomicrobia, Methanonatronarchaeia, Halobacteria

Because *bd*-type and HCO oxygen reductases are known to be subject to frequent transfer by LGT, we tested whether our sample produces similar results as previous studies [40,41,66,68,69,71]. For each reductase we generated a protein tree based on the best blast hits from the balanced RefSeq dataset. The leaves of the protein trees are colored according to their affiliation to groups, representing possible origins in the time tree and were checked whether they are monophyletic or not (Fig. 5, Supplemental Table 4). Reductases were defined as highly affected by LGT if the groups were mainly represented by several clades in the protein tree. In *bd*-type and HCO reductase protein trees, the groups per possible origin are widely spread and usually not monophyletic (Fig. 5a-b). Only three groups are monophyletic in the *bd*-type protein tree including Aenigmarchaeota, Thermococci and Chlamydiota (Fig. 5a, Supplemental Table 4). The HCO reductase protein tree has only one monophyletic group corresponding to the taxon Thermococci (Fig. 5b, Supplemental Table 4), which however contains a maximum of five strains, permitting no strong inference about monophyly.

Despite the small number of genomes and groups in the AOX protein tree, no monophyletic group is found (Fig. 5c). This suggests that the AOX reductase is also transferred via LGT in prokaryotes. However, the transfer of genes is restricted to Pseudomonadota. PTOX reductase do not seem to be affected by LGT. They are found only in Cyanobacteriota, making the protein tree a single monophyletic group (Fig. 5d). The current sample and analysis confirms previous reports for the massive role of LGT in the evolution of *bd*-type, HCO and AOX oxygen reductases [40,41,66,68,69]. One origin and 40 subsequent transfers for *bd* oxidases and one origin plus 32 transfers for HCOs inferred from the species trees (Figs. 2, 3) might seem like a large amount of LGT for oxygen reductases, but the number of transfers inferred from the enzyme phylogenies themselves (Fig. 5.ab) are 124 and 121 respectively, vastly exceeding the bare minimum of 40 (*bd*) or 32 (HCO) transfers needed to account for the lineage distribution of the enzymes.

## 4 Discussion

There is widespread agreement that the Great Oxidation Event (GOE) marked the persistent accumulation of O_2_ in Earth’s atmosphere, as documented by several lines of geologic evidence [1,36]. In particular, the onset of the GOE is temporally constrained to ca. 2.32–2.22 based on the irreversible disappearance of mass-independently fractionated sulfur isotopes from the sedimentary record [101–103], interpreted as signaling a rise in atmospheric O_2_ > 10^−6^ of present atmospheric levels (PAL) [36]. While oxygenic photosynthesis necessarily evolved prior to the GOE, the oldest body fossils interpreted as Cyanobacteria only appear ca. 1.9 Ga [104], leaving geochemical reduction-oxidation (redox) proxies as the primary tools for resolving when environmental O_2_ – and, by extension, oxygenic phototrophs – first appeared in Earth’s surface environment [1].

Numerous geochemical studies reporting the concentrations of redox sensitive metal concentrations and metal isotope ratios of sedimentary rocks have inferred that oxygenic photosynthesis predated the GOE by up to ca. 600 million years [29,105–107]. Geochemical and mineralogical data associated with the morphology of lacustrine stromatolites have also been used as evidence for oxygenic photosynthesis by ca. 2.7 Ga [108,109]. The conclusion that oxygenic photosynthesis significantly predated the GOE has inspired numerous efforts to explain how photosynthetic O_2_ production could have operated on Earth for hundreds of millions of years without oxygenating the atmosphere [110]. The proposed mechanisms vary, but tend to emphasize either enhanced O_2_ sinks, such as O_2_-consuming reactions with marine and atmospheric reductants [2,36], or diminished O_2_ sources, namely extrinsic or intrinsic caps on cyanobacterial primary production, from phosphorus limitation [111], Fe^2+^ toxicity [112], nitrogenase inhibition by O_2_ pre-GOE [113], to low metabolic efficiencies [114]. Despite the ever-growing list of these proposed mechanisms, no clear consensus exists on which one (or combination) of these—if any— actually works as an explanatory platform for advocating for an early origin of oxygenic photosynthesis relative to the GOE.

Although a minority view [36], the simplest explanation for why the GOE happened when it did and not earlier is that oxygenic photosynthesis originated in cyanobacteria only shortly before the GOE [1], and that the rapid rise in O_2_ at the GOE simply reflects the rapid (initially exponential) growth of cyanobacteria subsequent to their origin [59]. Collectively, geochemical evidence for free O_2_ before the GOE has been criticized as reflecting post-depositional alteration with oxic waters [9,30], and as involving light-driven redox reactions that occurred in the absence of free O_2_ [37,39]. Other geochemical evidence from shallow-water banded iron formations has been used to argue that the marine surface and atmosphere contained <10^−6^ PAL O_2_ ca. 2.45 Ga, implying that oxygenic photosynthesis had not yet evolved by this time [115]. According to a simple box model, photosynthetic oxygen production could have potentially overwhelmed atmospheric and marine O_2_-sinks (e.g., atmospheric H_2_ and marine Fe^2+^) within ca. 100,000 years of its origin [116].

Together, the idea that oxygenic photosynthesis originated only shortly before the GOE arguably represents the simplest and most straightforward reading of the geologic record in the absence of 1) unequivocal evidence for free O_2_ (and oxygenic phototrophs) prior to the GOE, and 2) a satisfying explanation for how photosynthetic O_2_ production could have operated for over a half-billion years with oxygenating the atmosphere.

Many reports infer the presence of oxygen in earth history from molecular phylogenetic studies [13,19–23], starting with the early study by [117]. Inferences of oxygen in Earth history from gene trees remain contentious because the use of molecular clocks is inapplicable if the gene in question has been affected by lateral gene transfer. All prokaryotic genes have been affected by LGT [26], in particular genes involved in oxygen metabolism [16]. In a molecular clock study, LGT systematically pushes the age of the gene in question artefactually deep, towards the root of the tree. Here we have taken the converse approach in that we allow LGT freely, we use geochemical evidence for the global appearence of oxygen at the GOE as a calibration point for the age of oxygen-dependent respiration, and we plot the appearance of oxygen reductases on a phylogenetic tree constructed from the ATP synthase, a largely vertically inherited gene [81]. The tree that we have used for plotting oxygen reductases was constructed by others as a general timeline reference for prokaryotic evolution, independent of oxygen reductase evolution.

As outlined before, there are isolated reports that trace amounts of oxygen might be synthesized from various reactions prior to the GOE, but these reports are controversial and do not mesh with the evidence for the existence of the GOE [12–16,18]. There are also claims for the occurrence of whiffs of oxygen prior to the GOE [7,8], but the samples in question could have been oxidized post-sedimentation [9], a finding that was rebutted [10] with rebuttal [11] in return. Our reading of the geochemical record is consent with the conservative and straightforward interpretation that the GOE represents the first global appearance of oxygen in Earth history [1,9,102]. We thus interpret the GOE as the earliest time point at which functional O_2_ reductases could have arisen. We also assume that LGT occurred freely in the evolution of oxygen reductase genes, consistent with earlier studies [40–42,66,68,69,97] and with the trees of oxygen reductases presented here (Fig. 5). With these simple premises, we find that *bd* oxidase and HCO gene evolution fit more or less perfectly with an origin of oxygen reductases at the GOE, followed by subsequent transfers to different lineages throughout the low oxygen phase of evolution called the Pasteurian billion, because Earth’s atmospheric O_2_ content was close to the Pasteur point (the O_2_ concentration at which facultative anaerobes switch to O_2_ respiration) during that time (Fig. 4). The present data do not indicate which lineage invented *bd* oxidases (or HCO), but given the number of subsequent transfers involved, the identity of the *bd*- and HCO-inventing lineages does not impact our findings.

One could argue that Cyanobacteria were the first organisms to evolve oxygen reductases, because they were the first to be confronted with O_2_, namely that produced by water-splitting photosynthesis [71]. However, O_2_ diffuses out of the cyanobacterial cell faster than it is produced, such that the O_2_ concentration in cyanobacterial cells generated by de novo O_2_ production is 0.25 μM to 0.025 μM [118]. The O_2_ from endogenous production is thus roughly 1000 fold lower than modern concentrations, and well within the Km range of *bd* and HCO enzymes (10 nM to 10 μM, [79]), and sufficient to support the origin of oxygen reductases in cells other than cyanobacteria in Earths’ gradually oxygen-accruing environment. As a result, oxygen reductases could have arisen, in principle, in any heme-producing lineage with a preexisting anaerobic respiratory chain.

Prior to the GOE, Earth was inhabited by anaerobes [119]. O_2_ is inhibitory for many anaerobes in that it is a stable diradical that can, however, readily accept single electrons from one-electron donors such as quinols, flavins and in particular FeS clusters to generate the ${\text{O}}_{2}^{•-}$ superoxide radical, a highly reactive oxidant and toxic reactive oxygen species (ROS) [61,120–122]. While flavins, quinols and other cofactors including thiamin [123] generate toxic ROS, they remain active as co-factors upon contact with O_2_. By contrast, many FeS clusters undergo oxidative damage upon contact with O_2_, such that O_2_ inactivates enzymes with surface accessible FeS clusters [61]. Note, however, that many FeS clusters are stable in the presence of O_2_, for example the eight FeS clusters in complex I of the mammalian respiratory chain [124]. It has been suggested that the initial function of oxygen reductases, especially *bd*-type oxidases, was to keep the cytosol free of O_2_ [125,126], yet for O_2_ detoxification, most cells possess dedicated, soluble oxygen-removing and ROS detoxification enzymes, including NADH oxidases and superoxide dismutases [16,19,121,127,128]. In the wake of the GOE, *bd*-type and HCO oxidases could assume their roles in energy conservation, functioning in aerobic respiration in some lineages, in denitrification in others, and in some cases, functioning in biosynthetic pathways [40–42,67].

### 4.1 The Lomagundi (or Lomagundi-Jatuli) excursion

**Fig. 6:**
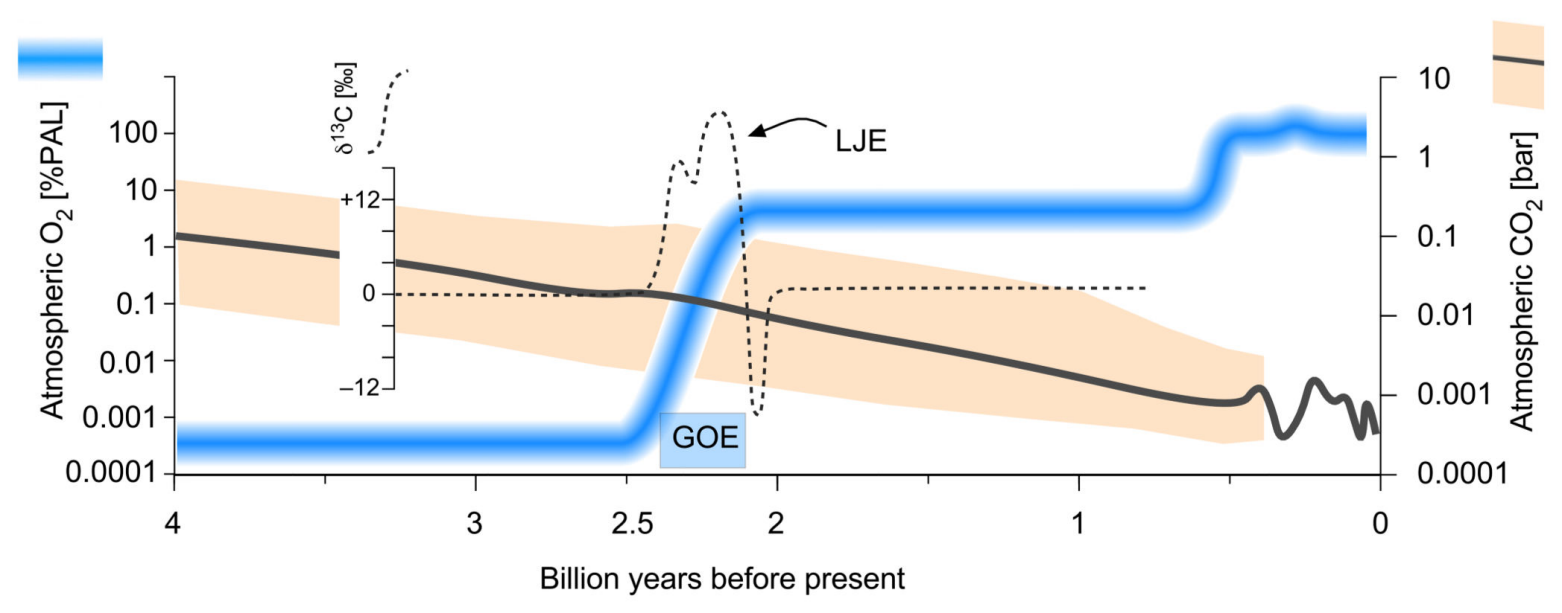
Atmospheric O_2_ and CO_2_ during the last 4 billion years in comparison to δ^13^C values including the Lomagundi-Jatuli excursion (LJE) and the Great oxidation event (GOE). Comparison of the evolution of δ^13^C values (dashed line, [3]), O_2_ values (blue line, [60]) and CO_2_ values (gray line, [36]) during the last 4 billion years. (For interpretation of the references to colour in this figure legend, the reader is referred to the web version of this article.)

An aspect of O_2_ history that has not been previously addressed by molecular studies is the Lomagundi excursion. More or less concomitant with the GOE, there is a ^13^C isotope anomaly in the geochemical record called the Lomagundi or Lomagundi-Jatuli excursion [3,48] that designates a ^13^C enriched marine dissolved inorganic carbon (DIC) pool, which is the sum of dissolved CO_2_, ${\text{HCO}}_{3}^{-}$ and ${\text{CO}}_{3}^{2-}$ (Fig. 6). This increase in ^13^C in the DIC pool indicates increased primary production by oxygenic photosynthesizers, because Rubisco discriminates against ^13^CO_2_, preferentially incorporating ^12^CO_2_ into biomass [129], leaving excess ^13^C in the atmosphere and hence in the DIC pool. Forests during the Carboniferous, for example, deposited CO_2_ as photosynthate that became rapidly buried and thus became our modern coal reserves, generating atmosphere O_2_ levels on the order of 150 % PAL, which is reflected in high ^13^C vaules in DIC of the Caboniferous. Today, photosynthetic CO_2_ fixation and O_2_ respiration take place are roughly equal rates, such that atmospheric O_2_ levels are stable [64,65]. It is now agreed that the high ^13^C at the Lomagundi excursion need not reflect O_2_ levels vastly exceeding the present value of 21 % *v*/v [48], but the causes for the appearance and disappearance of the Lomagundi are still debated. Very complicated, multifactorial whole-ecosystem models have been proposed as a cause of the LJE [130] but without identification of specific processes underlying the isotopic excursion. Recent studies have investigated the possibility that Rubisco ^13^C discrimination might have been higher in the ancient past [131,132] by investigating the discrimination properties of ancestral Rubisco enzymes, but the measured effects were small, also in the presence atmospheres containing 2–5 % CO_2_, which likely existed around the time of the GOE [36]. Altered properties of ancient Rubisco enzymes are, in principle, a possible cause of the LJE, as are a number of other factors, as outlined by Prave [133].

**Fig. 7:**
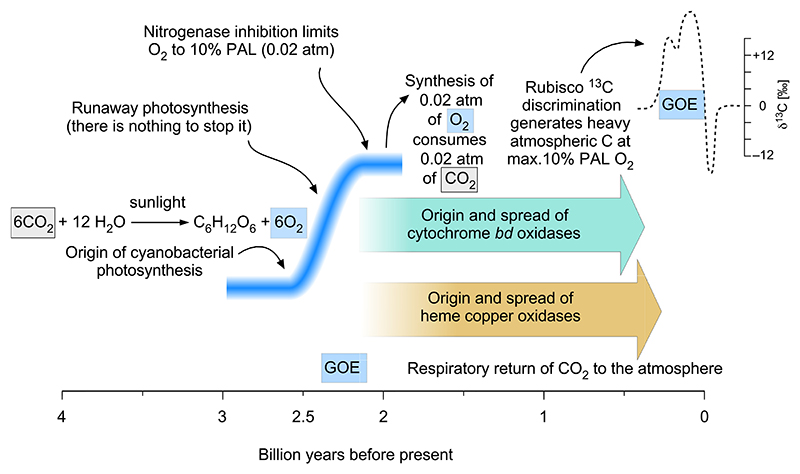
Model for the causes of Lomagundi-Jatuli excursion (LJE) in connection with the evolution of atmospheric gases as O_2_ and CO_2_.

We consider a sequence of simple processes with few variables at the origin of the LJE, as outlined in Fig. 7. Reading the geochemical record with Occam’s razor, there was no cyanobacterial O_2_ production prior to the GOE. With the origin of water-splitting photosynthesis, cyanobacteria produced an atmosphere of roughly 2 % oxygen by the end of the LJE and the end of the GOE. There is no explanation in the geochemical record why oxygen stayed flat during the Pasteurian era and nothing existed that limited cyanobacterial growth. However oxygen accumulation ceased at ~2 % and did not exceed ~2 % because nitrogenase is inhibited by 2 % O_2_, and without nitrogenase, no net CO_2_ fixation (cyanobacterial cell synthesis) is possible [58,59].

Note that nitrogenase is not inhibited by endogenous O_2_ production, because O_2_ rapidly diffuses out of the oxygen-producing cell, such that endogenous O_2_ synthesis generates intracellular O_2_ levels of 0.25 μM to 0.025 μM [118], 10 to 100 times lower than that required to inhibit nitrogenase [59]. In oxygenic photosynthesis, one CO_2_ is consumed for every O_2_ produced. The GOE would have consumed all CO_2_ contained in a 2 % CO_2_ atmosphere. Even with a modern Rubisco, that CO_2_ depletion would be expected to generate a very substantial alteration in the ^13^C isotope record reflecting high carbonate ^13^C simply as evidence of increased carbon burial [48,129]. If the atmosphere contained less than atm CO_2_ at the time of the LJE (Fig. 7) [36], the GOE (which generated 0.02 atm O_2_ in the atmosphere) would have essentially scrubbed the atmosphere free of CO_2_, bringing O_2_ production to a halt, which apparently did not happen (Fig. 7). A 5 % CO_2_ atmosphere would have been depleted in CO_2_ roughly by half.

One could argue that respiratory processes were replenishing atmospheric CO_2_ levels as soon as carbon burial at the GOE commenced. But according to the age of oxygen reductases that we have estimated here, oxygen respiration had either not yet evolved at all at the GOE or had not yet become widespread among bacterial lineages (Fig. 4). In the absence of *bd* oxidases or HCO in respiratory chains, anaerobic respirations could have returned some CO_2_ to the atmosphere. But by the measure of modern CO_2_ cycling, the contribution of anaerobic respirations (SO_2_, Fe^3+^) or fermentations would have been modest [64,65], because more than 99 % of biological CO_2_ production today comes from O_2_ respiration.

The end of the LJE is marked by a sharp spike of low ^13^C, suggesting, in standard models, rapid release to the DIC pool of sequestered ^12^C-rich organic material—derived from cells of the newly arisen cyanobacterial lineage in this model. We propose that this rapid release of sequestered organic carbon at the end of the LJE corresponds to the origin of *bd* and heme-copper oxygen reductases and the respiration of a substantial portion of light carbon buried during the GOE. Oxygen levels did not react to the origin and spread of oxygen reductases because nitrogenases imposed an upper on O_2_-levels independent of oxygen consumption [58,59].

In this proposal, the LJE indicates a sharp increase in carbon burial at a level sufficient to generate a ^13^C enrichment in the marine DIC pool, but at no more than 2 % O_2_ in the atmosphere, because of nitrogenase inhibition. Furthermore, this proposal entails neither massive export of the greenhouse gas methane to the atmosphere [130], nor does it entail the formation of an ozone layer [130], which under standard models arose long after the GOE, about 600 MY ago [39,134]. Our model requires no attributes of oxygenic photosynthesis or cyanobacterial Rubisco that differ from modern. It does however require an atmospheric CO_2_ level (0.02 atm) sufficient to support the synthesis of 0.02 atm of O_2_. Following the origin of oxygen reductases at the end of the LJE and the GOE, CO_2_ production through respiration and O_2_ production through cyanobacterial photosynthesis could have fallen into quantitative balance, as in the modern carbon cycle [64], but in an atmosphere of constant ~2 % O_2_ for almost 2 billion years until the origin of land plants [49], because of nitrogenase inhibition [58,59] by O_2_.

Supplementary data to this article can be found online at https://doi.org/10.1016/j.bbabio.2025.149575.

## Supplementary Material

Supplemental Fig.

Supplemental Table 1

Supplemental Table 2

Supplemental Table 3

Supplemental Table 4

## Data Availability

Data will be made available on request.
