# Supplementary material for: Oxygen reductase origin followed the great oxidation event and terminated the Lomagundi excursion
Source: Biochim Biophys Acta Bioenerg. Author manuscript; Available in PMC 2026 Jan 30. (PMC7618691; doi:10.1016/j.bbabio.2025.149575)
Supplement: Supplemental Fig. [file EMS211948-supplement-Supplemental_Fig_.docx]

**Supplemental Data**

**Supplemental Figure 1. Presence of heme biosynthesis pathways in Methanogens and Halobacteria.** Proportion of strains in taxon (y-axis) per protein of the siroheme-, the protoporphyrin- and the coproporphyrin-dependent heme biosynthesis pathway (x-axis). Taxa marked with an asterisk (*) correspond to Methanomicrobiales.

**Supplemental Figure 2. Presence and absence of VhtACG and HdrDE protein complexes in Methanogens and Halobacteria.** Presence (black) or absence (white) of proteins corresponding to protein complexes VhtACG and HdrDE that are involved in the energy conservation of Methanosarcinales and are known to contain cytochrome *b*.

**Supplemental Figure 3. Occurrence of AOX and PTOX oxygen reductase in a dated tree of life.** Branches in the dated tree of life obtained from Mahendrarajah et al. (2023) are colored according to the presence of AOX (red) and PTOX (gree) or absence (gray) oxygen reductases. Eukaryotes were not included in the analysis and are therefore colored in higher gray tones, as are taxa that were not present in the comparative dataset. Dark red dots at nodes represent possible origins of AOX oxygen reductase and dark green dots possible origins of PTOX. Purple bars represent the percentage of strains within the taxa that have reductases. In this cyanobacterial sample only one AOX was found and therefore not colored in red since Cyanobacteria (and plastid) have a different alternative oxidase di-iron, named PTOX, which is widespread in plants.

**Supplemental Figure 4. Distribution and of ages per group on geological timescale.** Distribution of ages per possible origin (group) within the dated tree of life for the AOX (red) and PTOX (green) oxygen reductase. The age [million years ago] per group is shown on the x-axis and the corresponding kernel density function (KDE) is placed over the corresponding distribution.
